# Effects of Dietary Protein and Fat Levels on Growth Performance, Nutrient Digestibility, Serum Indexes, and Rectal Fecal Microbiota of Sika Deer (*Cervus nippon*) Fawns in Early Wintering Period

**DOI:** 10.3390/ani15070908

**Published:** 2025-03-21

**Authors:** Zuer Gao, Jiaxin Tian, Qiaoru Zhang, Haoran Sun, Qingkui Jiang, Tietao Zhang

**Affiliations:** 1 Institute of Special Animal and Plant Sciences, Chinese Academy of Agriculture Sciences, Changchun 130112, China; gze1010@163.com (Z.G.); tjx0813@126.com (J.T.); sarahzhang96@163.com (Q.Z.); solomoncat@163.com (H.S.); 2 Public Health Research Institute, New Jersey Medical School, Rutgers Biomedical and Health Sciences, Rutgers, The State University of New Jersey, Newark, NJ 07103, USA

**Keywords:** young sika deer, protein level, fat level, growth performance, nutrient absorption, serum biomarkers, fecal microbiome

## Abstract

This study examined the effects of dietary crude protein (CP: 18%, 15%) and crude fat (EE: 8%, 4%) levels, and their interactions, on growth performance, nutrient digestibility, serum indices, and rectal fecal microbiota in sika deer fawns during early wintering. A two-month 2 × 2 factorial experiment was conducted using 32 healthy five-month-old male fawns randomly assigned to four groups: P18E8 (18% CP, 8% EE), P18E4 (18% CP, 4% EE), P15E8 (15% CP, 8% EE), and P15E4 (15% CP, 4% EE). The P18E4 group showed the highest total weight gain and average daily gain (*p* < 0.05), along with greater apparent digestibility of dry matter, crude protein, calcium, and fiber fractions (*p* < 0.05). Serum urea content was significantly lower in this group, indicating improved nitrogen utilization (*p* < 0.05). Dominant fecal microbiota at the phylum level across all groups included Firmicutes_A and Bacteroidota, with the P18E4 group showing a unique genus composition within *Bacteroidota*, known for enhancing fiber digestion. In summary, a diet with 18% CP and 4% EE optimized growth performance, nutrient digestibility, and gut microbiota composition, providing a strategy for improving the health and productivity of sika deer fawns during overwintering.

## Simple Summary

Feeding young sika deer the right balance of protein and fat is crucial for their growth and health. In this study, 32 young male sika deer were divided into four groups and fed different diets for two months to determine how protein and fat levels affect their development. The results showed that deer fed a diet with 18% protein and 4% fat grew faster, had better digestion of key nutrients, and showed improved health markers in their blood compared to the other groups. Additionally, this group exhibited enhanced gut microbiota. These findings suggest that a diet with 18% protein and 4% fat supports healthy growth, nutrient absorption, and gut health in young sika deer, helping them thrive during early winter. This research provides valuable insights for improving feeding strategies in deer farming, promoting better animal health and productivity.

## 1. Introduction

The sika deer (*Cervus nippon*) has a long history of domestication and artificial breeding across the globe. Renowned for their medicinal and economic value, sika deer products hold significant importance [1], with pilose antlers historically serving as the cornerstone of the sika deer breeding industry. However, over the past decade, the global demand for meat, combined with the recognized nutritional benefits of venison, has driven a growing interest in deer meat consumption [2]. The meat production performance of sika deer is a critical focus for sika deer fawn.

The young deer stage, from birth to 18 months, significantly influences the meat production performance of sika deer in adulthood, as it represents the fastest growth and development period for sika deer. After weaning, sika deer fawns primarily rely on dietary nutrients to support their growth and development. During this period, their growth rate, although slightly reduced compared to the lactation phase, can achieve an average daily gain of 200–240 g under optimal feeding conditions [3]. Growth in young deer involves muscle development and fat accumulation, as well as changes in the fecal microbiome [4]. Nutritional demands are particularly high during the winter months, as young deer need additional nutrients to sustain basal growth and metabolic needs. Therefore, the adequacy and balance of dietary nutrition directly affect the fecal microbiota composition and growth performance of sika deer fawns during the wintering period.

Protein and fat are critical components of the sika deer diet. Protein forms the structural basis of tissues and supports rapid growth, while fat serves as an essential source of energy for animals. Studies have shown that 2% dietary fat significantly improved the digestibility of crude protein, crude fat, calcium, and phosphorus in 3-year-old sika deer compared to higher fat levels [5]. Protein demands are particularly high for sika deer fawns due to their rapid growth and heightened protein metabolism. However, excessive dietary protein levels –can exert detrimental effects on sika deer [6]. Increased protein availability influences rumen microbial populations by promoting the proliferation of ammonia-producing bacteria, such as *Clostridium sticklandii* and *Peptostreptococcus anaerobius* [7,8], which generate ammonia (NH_3_), elevating ruminal pH due to its weakly basic nature [9]. Elevated ammonia levels, in turn, support the growth of ammonia-utilizing, fiber-degrading bacteria like *Fibrobacter succinogenes*, *Ruminococcus albus*, and *Ruminococcus flavefaciens*, which require ammonia for protein synthesis [10]. A higher ruminal pH (6.2–7.0) further favors cellulolytic bacteria while reducing acid-tolerant species such as *Streptococcus bovis* and *Lactobacillus spp.* [11,12]. The microbial imbalances can impair animal health, reduce feed efficiency, and induce animal growth [13,14].

Gao et al. found that an 18% crude protein level in concentrate supplement was optimal, with each deer requiring 208 g of crude protein, 146 g of digestible crude protein, and 117 g of metabolizable protein daily [15]. Extensive research on various ruminants has also highlighted the interplay between energy and protein as co-limiting factors influencing growth and production [16,17]. However, the specific effects of protein and fat interactions on sika deer remain largely unexplored.

This study aimed to comprehensively evaluate the effects of dietary protein and fat levels on the growth performance, nutrient digestibility, serum indexes, and rectal fecal microbiota of sika deer fawns. The findings provide a theoretical basis for improving the meat production performance of sika deer and establishing a rational cultivation system for the meat-producing sika deer.

## 2. Materials and Methods

All animal experimental procedures were approved by the Animal Ethics Committee of the Chinese Academy of Agricultural Sciences and performed according to the guidelines for animal experiments of the National Institute of Animal Health (NO. ISAPSAEC-2023-037SD).

The experiment was carried out in Dong ’ao deer breeding base, Shuangyang District, Changchun City, Jilin Province, China. Thirty-two 5-month-old healthy male sika deer fawns with similar body weights were randomly divided into 4 groups with 8 replicates per group and 1 deer per replicate. Animals were housed in an intensive (pen-based) farming system, with eight sika deer per group housed in a pen, providing a space allowance of approximately 5–10 m^2^ per deer. The temperature ranged from −20 °C to −15 °C throughout the experiment period. Animals had free access to water and feed, and regular cleaning and waste management were conducted to maintain hygiene. Four groups of total mixed ration (TMR) diets were prepared using a 2 (CP: 15%, 18%) × 2 (EE: 4%, 8%) two-factor crossover design: sika deer fawns were fed with diets composed of 18% CP and 8% EE (P18E8), 18% CP and 4% EE (P18E4), 15% CP and 8% EE (P15E8), or 15% CP and 4% EE (P15E4). The TMR diet was based on corn, corn germ meal, distillers dried grains with soluble (DDGS), alfalfa meal, salt, and premix, with soybean meal as the main protein source and soybean oil as the fat source. A 7-day adaptation trial was conducted to acclimate the animals to the experimental diets and estimate their feed intake. During the 60-day early wintering experiment (15 November 2023, to 15 January 2024), animals were offered an adequate amount of the TMR diet based on the estimated feed intake at 7: 30 and 15: 30 daily, with ad libitum access to drinking water (Table 1). Feed intake were determined by recording the amount of feed provided and subtracting the leftovers.

### 2.1. Weight Determination and Feed Intake

Initial weight (IW) and final weight (FW) of animals were recorded at day 1 and day 60 before feeding. During the test, feed intake was recorded on a daily basis.

Total weight gain (TWG) = FW − IW

Average daily weight gain (ADWG) = TWG/60

Average daily feed intake (ADFI) = total feed intake/60

Feed-to-weight ratio (F/W) = ADFI/(ADWG × 10^3^)

### 2.2. Sample Collection and Chemical Analysis (Diet Ingredients and Excreta)

Samples of each diet were collected and analyzed for dry matter (DM), crude protein (CP), ether extract (EE), crude fiber (CF), calcium (Ca), phosphorus (P), neutral detergent fiber (NDF) and acid detergent fiber (ADF) according to the methods described in AOAC 2007 [18]. The gross energy (GE) concentration was measured using an adiabatic bomb calorimeter (IKA C2000; IKA company, Staufer, Germany).

Fecal samples were collected prior to morning feeding on day 58, day 59, and day 60 according to the partial collection method, and acid-insoluble ash was used to estimate total fecal output [19]. Briefly, on each of the three days, approximately 100 g of fresh fecal samples were collected from each animal immediately after excretion. Visible contaminants such as deer hair and gravel were removed, and 10% dilute sulfuric acid was sprayed onto the samples to fix nitrogen. The samples were then sterilized at 85 °C for 2 h, followed by drying at 65 °C to a constant weight. Dried samples were crushed and sieved through a 40-mesh sieve to prepare air-dried samples for subsequent analyses of CP, EE, Ca, P, NDF, and ADF. The apparent digestibility of nutrients was calculated using the following formula:

Apparent digestibility (%) = 100 − (100 × A/A1 × B1/B),

where A is the percentage of 2 mol/L hydrochloric acid insoluble ash in the diet, A1 is the percentage of 2 mol/L hydrochloric acid insoluble ash in the feces, B1 is the percentage of the nutrient in the feces, and B is the percentage of the nutrient in the diet.

### 2.3. Serum Collection and Analysis

On the last day of the experiment, animals were anesthetized, and the jugular vein blood was collected into a procoagulant tube before morning feeding. The blood sample was centrifuged at 3500 rpm for 10 min, and the supernatant was taken and stored in a 1.5 mL EP tube at −80 °C for testing.

Triglyceride (TG), cholesterol (CHO), high-density lipoprotein (HDL), lo- density lipoprotein (LDL), total protein (TP), albumin (ALB), glucose (GLU), and urea contents use kits from Biosino Biotechnology and Science Co., Ltd. (Beijing, China), and were determined by Wetu-E biochemical analyzer in strict accordance with the instructions of the reagents.

The contents of growth hormone (GH) and insulin-like growth factor (IGF) in deer were determined by ELISA. The kits were purchased from Jiangsu Fiya Biotechnology Co., Ltd. (Yancheng, China).

### 2.4. Rectal Fecal Sample Collection for Microbiome Analysis

To analyze the fecal microbiome, sika deer fawns were anesthetized before the morning feeding on the final day of the experiment. Approximately 5–10 g fecal samples were collected from the anal region of each fawn using sterile gloves and placed into sealed 5 mL sterile centrifuge tubes. The samples were immediately frozen in liquid nitrogen and subsequently stored at −80 °C for microbiota composition analysis.

### 2.5. DNA Extraction, PCR Amplification, and Sequencing of Gastrointestinal Microbiota

Total genomic DNA samples were extracted using the OMEGA Soil DNA Kit (M5635-02) (Omega Bio-Tek, Norcross, GA, USA), following the manufacturer’s instructions, and stored at −20 °C prior to further analysis. The quantity and quality of extracted DNAs were measured using a Nanodrop NC2000 spectrophotometer (Thermo Fisher Scientific, Waltham, MA, USA) and agarose gel electrophoresis, respectively. PCR amplification of the bacterial 16S rRNA genes V3–V4 region was performed using the forward primer 338F (5′-ACTCCTACGGGAGGCAGCA-3′) and the reverse primer 806R (5′-GGACTACHVGGGTWTCTAAT-3′).

The library was constructed using Illumina’s TruSeq Nano DNA LT Library Prep Kit. Two-end sequencing was performed on the Illumina NovaSeq instrument for the qualified library. The biological information of the microbiome was analyzed using QIIME2 2022.11. The original sequence data uses DADA2 to perform data processing such as quality filtering, denoising, splicing, and chimera removal on the sequence. The sequences obtained above were clustered into Operational Taxonomic Units (OTUs) by default with 97% consistency, and the representative sequences of OTUs were screened for species annotation analysis (threshold was set to 0.8). Alpha diversity analysis The QIIME2 software was used to calculate the following five diversity indexes for each sample, including the Chao1 index, Observed species index, and Shannon index, and to compare the abundance of OTUs between different samples. The linear discriminant analysis effect size (LEfSe) method was used to detect the classification units with rich differences between groups.

### 2.6. Statistical Analyses

Data were analyzed using SPSS software (IBM SPSS Statistics 27; IBM-SPSS Inc., Chicago, IL, USA) and represented as mean ± SE. A two-way ANOVA was used to analyze the effects of dietary protein and fat levels and their interactions on growth performance, nutrient digestibility, and serum indexes of sika deer fawns in the early wintering period. *p* < 0.05 indicated a significant difference. When there was a significant interaction between protein and fat, a one-way ANOVA was performed for all data, and Duncan’s test was used for multiple comparisons to analyze the significance among treatment groups. The above criteria (w, x, y, z) (*p* < 0.05), and the effect of a single influencing factor were not considered. When there was no significant interaction between protein and fat, the protein or fat level was fixed, then the effect of the other factor was analyzed: when the fat level was fixed, the effect of protein level was analyzed, the above standard (a, b) indicated a significant difference between treatment groups (*p* < 0.05); when the protein level was fixed, the effect of fat level was analyzed, the above standard (A, B) indicated a significant difference between treatment groups (*p* < 0.05).

## 3. Results

### 3.1. Effects of Dietary Protein and Fat Levels on Growth Performance of Sika Deer Fawns

As shown in Table 2, dietary protein and fat levels, along with their interactions, significantly influenced the body weight of sika deer fawns during the early wintering period, but no significant effects were observed on feed intake. Initial and final body weight did not differ among the four experimental groups. The F/W was significantly lower in the 18% protein groups compared to the 15% protein groups (*p* < 0.05). No significant differences were observed in TWG, ADG, or F/W between the two fat levels. However, dietary protein and fat levels showed significant interactions affecting TWG, ADG, and F/W (*p* < 0.05). The P18E4 group exhibited significantly higher TWG and ADG than the other three groups, while the F/W of the P15E4 group was significantly higher than that of the other groups.

### 3.2. Effect of Dietary Protein and Fat Levels on Nutrients Apparent Digestibility of Sika Deer Fawns

Table 3 illustrates that dietary protein, fat levels, and their interactions significantly impacted the apparent digestibility of nutrients of sika deer fawns during the early wintering period. Digestibility of DM, CP, EE, Ca, P, NDF, and ADF was significantly higher in the 18% protein groups compared to the 15% protein groups (*p* < 0.05). The digestibility of EE and P was significantly greater in the 8% fat groups than in the 4% fat groups (*p* < 0.05), whereas the digestibility of Ca, NDF, and ADF was significantly lower in the 8% fat group (*p* < 0.05). CP digestibility remained unaffected by fat levels. Notably, significant interaction between protein and fat levels was observed for the digestibility of DM, CP, Ca, NDF, and ADF (*p* < 0.05). The P18E4 group had significantly higher digestibility of DM, CP, Ca, NDF, and ADF than the other three groups (*p* < 0.05).

### 3.3. Effect of Dietary Protein and Fat Levels on Serum Indices of Sika Deer Fawns

Table 4 shows that dietary protein, fat levels, and their interactions significantly affected serum CHO, HDL-C, and urea concentrations during the early winter period, but no significant effects were found on other biochemical indices. HDL-C and urea levels were significantly lower in the 18% protein level groups compared to the 15% protein groups (*p* < 0.05), with no significant differences in CHO content. Conversely, CHO, HDL-C, and urea were significantly higher in the 8% fat groups than in the 4% fat groups (*p* < 0.05). Significant interactions were observed between dietary protein and fat levels for CHO, HDL-C, and urea (*p* < 0.05). CHO and HDL-C levels in the P15E8 group were significantly higher than in the other groups (*p* < 0.05), while urea levels in the P18E4 group were significantly lower (*p* < 0.05).

Table 5 indicates that dietary protein and fat levels, as well as their interactions, significantly influenced serum GH levels, but did not affect IGF levels. GH levels were significantly higher in the 18% protein groups than in the 15% protein groups (*p* < 0.05), and significantly higher in the 8% fat level groups than in the 4% fat groups (*p* < 0.05). There was a significant interaction between dietary protein and fat levels on GH content (*p* < 0.05). The P18E8 group exhibited significantly higher GH levels than the other groups (*p* < 0.05).

### 3.4. Effects of Dietary Protein and Fat Levels on Rectal Fecal Flora of Sika Deer Fawns

**Figure 1:**
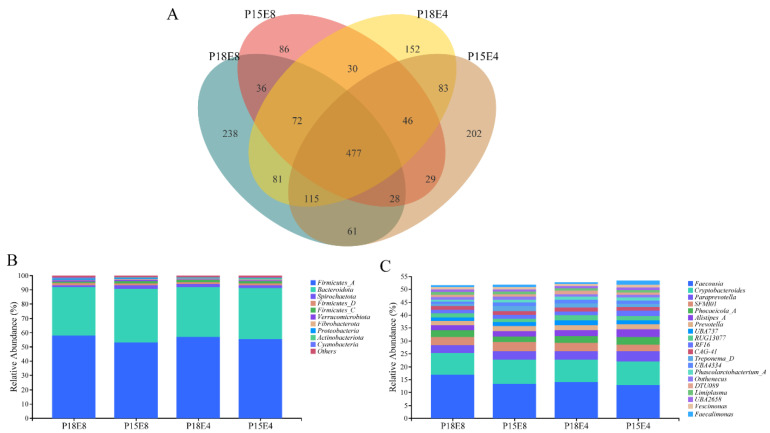
Venn diagram and taxonomic composition diagram of rectal fecal microbiota. (**A**) Venn diagram of rectal fecal microbiota. (**B**) Abundances of top 10 bacteria at phylum level on rectal fecal microbiota. (**C**) Abundances of top 20 bacteria at genus level on rectal fecal microbiota.

Illumina Nova sequencing of rectal fecal samples identified 1736 operational taxonomic units (OTUs) at a 97% consistency threshold. As shown in Figure 1A, 477 OTUs are shared among the four groups. Taxonomic annotation using QIIME2 (2019.4) revealed that Firmicutes_A and Bacteroidota were the dominant bacteria at the phylum level in all groups (Figure 1B), while the dominant genera included *Faecousia*, *Cryptobacteroides, Paraprevotella, SFMI01*, and *Phocaeicola_A* (Figure 1C).

Alpha diversity analysis revealed significant differences among the groups (Table 6). Groups with higher protein levels exhibited a significantly higher Shannon index (*p* < 0.05) and showed an increasing trend in the Chao1 index (*p* = 0.90) and observed species index (*p* = 0.70) compared to the low-protein groups. These findings suggest that higher dietary protein intake enhances both the richness and diversity of bacterial communities in sika deer fawns.

**Figure 2:**
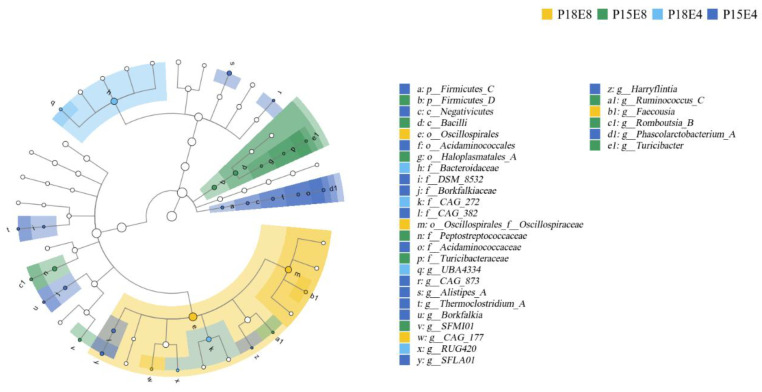
Linear discriminant analysis effect size (LEfSe) analysis on rectal fecal microbiota of sika deer fawns in early wintering period. The node size corresponds to the average relative abundance of the taxonomic unit. Hollow nodes represent taxonomic units with no significant differences between groups, while colored nodes indicate significant differences between groups. Letters label the taxonomic units that show significant differences between groups.

LEfSe diagram identified 31 bacteria with significant differences across the four groups from the phylum level to the genus level (Figure 2). *Oscillospirales* and *Oscillospiraceae* were significantly enriched In the P18E8 group, while *Bacteroidaceae* and *CAG_272* were significantly enriched in the P18E4 group (Figure 2).

## 4. Discussion

The appropriate dietary nutrition levels of sika deer fawns during the early overwintering period remain largely unexplored. This study contributes to this understudied area by evaluating the effects of varying protein and fat levels on body weight, nutrient digestibility, serum indicators, and intestinal microbiota in early wintering sika deer fawns. The results provide novel insights into optimizing nutritional strategies for this economically important species.

### 4.1. Growth Performance

The study revealed that sika deer fawns fed a diet containing 18% protein and 4% fat (P18E4) exhibited significantly higher TWG and ADG compared to the other groups. Additionally, fawns in the P15E4 group exhibited significantly higher F/W. These findings indicate that a higher protein level in the diet promotes weight gain in sika deer fawns. This result aligns with previous research showing that low-energy high-protein diets increase the average daily gain of dear during the antler growth period [20]. This is likely because sika deer fawns primarily rely on dietary to meet their growth requirements [21]. Similarly, studies on other ruminants have reported that higher dietary protein levels enhance growth. For example, calves fed high-protein diets from birth to 5 months of age exhibited increased body weight [22], while fattening lambs demonstrated significantly greater average daily gain [23]. Protein supports ruminant growth by stimulating muscle protein synthesis, modulating growth hormone levels, and enhancing rumen microbial diversity [24], which likely explains the superior growth performance observed in the P18E4 group. As sika deer feed traditionally does not include added oil, the effects of dietary fat on sika deer fawns were largely neglected, with studies examining the effects of dietary fat levels on the growth performance of sika deer fawns are lacking. We thus However, the four groups of feed in this experiment were supplemented with soybean oil. Dietary fat can enhance ruminant production performance to a certain extent [5]. Research on other ruminants has shown that fat supplementation can improve body weight gain, as seen in calves fed diets with 6% soybean oil, which achieved 9% greater weight gain than those on a control diet without added fat [25]. Fat serves as a vital energy source, yet excessive fat supplementation can adversely impact growth and digestive function. For instance, feedlot cattle have exhibited reduced growth performance with high levels of dietary fat [26]. In the present study, the effect of dietary fat on growth was minimal; however, the differences observed between the P18E4 and P18E8 groups indicate that higher fat levels may impede growth in sika deer fawns. In addition, as the animals were exposed to cold conditions, diets with lower heat increment (P15E4) can lead to more dietary energy being allocated to thermoregulation, potentially reducing energy available for weight gain [27]. These findings underscore the importance of achieving a balanced dietary composition. The P18E4 diet emerged as the optimal balance of protein and fat for promoting growth in early overwintering sika deer fawns, emphasizing the need for synergistic nutrient management to support optimal development.

### 4.2. Nutrient Digestibility

The P18E4 diet significantly enhanced the digestibility of DM, CP, Ca, NDF, and ADF, suggesting that improved nutrient digestion and utilization by the high-protein and low-fat diet of in sika deer fawns. High protein levels likely enhance ruminal fermentation and nutrient assimilation, as observed in other studies on ruminants [28,29]. Similar to the above results, the digestibility of nutrients in the 18% protein group was significantly higher than that in the 15% protein group. In contrast, higher fat levels appeared to suppress nutrient digestibility, likely due to adverse effects on rumen microorganisms of sika deer [5]. Previous studies reported that 2% dietary fat had significantly higher crude protein, crude fat, calcium, and phosphorus digestibility in 3-year-old sika deer compared to the 6% fat group [5]. Similarly, the apparent digestibility of ADF and NDF in the 8.7% rumen-protected fat group was significantly lower than that in the 4.8% rumen-protected fat group. These results emphasize the importance of balancing dietary fat levels to optimize nutrient digestibility and growth outcomes.

### 4.3. Serum Biochemical Indicators

The serum biochemical indexes of animals can reflect the metabolism, nutritional status, and disease of animals to a certain extent, thus indirectly reflecting the growth performance of animals. Serum indicators such as CHO, HDL-C, and urea nitrogen are significantly influenced by dietary protein levels. For instance, an increase in dietary protein level has been shown to reduce serum CHO in steers [30] and HDL-C in lambs [31]. However, the effects of dietary fat on these serum indices remain largely unexplored. In the present study, the serum CHO content of the 8% fat level group was significantly higher than that of the 4% fat level group, in accordance with previous findings that fat supplementation increases serum CHO levels in ruminants [32]. The 8% dietary fat also markedly increased the HDL-C in the serum of sika deer fawns. This is because HDL-C is mainly involved in lipid metabolism and transports CHO to the liver [33].

The P18E4 group exhibited significantly lower urea nitrogen levels, reflecting efficient nitrogen metabolism and reduced protein oxidation [34]. Similar to the above studies, this indicates that a high-protein and low-fat diet is beneficial to the intake of protein in the diet to promote the growth and development of sika deer fawns.

GH and IGF-1 are essential for skeletal and muscular development. Synthesis and release of GH and IGF-1 are closely influenced by the nutritional status of animals, with malnutrition shown to decrease serum IGF-1 and GH levels [35]. Our study advanced these results by reporting that dietary protein, fat levels, and their interaction significantly influenced serum GH levels. Notably, the P18E8 group exhibited higher GH content in the serum compared to other dietary groups. GH primarily stimulates growth through the production of IGF1 to promote its anabolic effects, such as skeletal and muscular development. However, the unchanged IGF1 level in the P18E8 group indicates that high GH probably has shifted metabolic priorities with respect to catabolic effects on fat metabolism [36] rather than promoting growth, which could explain the lack of increased body weight despite higher GH levels. These findings suggest that while high-protein and high-fat diets can influence GH levels, such diets may not be optimal for supporting the growth and development of sika deer fawns during the early wintering period. Striking a balance in dietary composition is essential to ensure the health and optimal development of these animals.

### 4.4. Intestinal Microbiota

The gut microbiome plays a pivotal role in nutrient digestion and absorption [37], host growth and development [38], and disease pathogenesis and progression [39]. The results of this study demonstrated that the number of OTUs in the rectal fecal flora of the P18E8 group was the highest, while the number of OTUs in the P15E8 group exhibited the lowest number. This suggests that dietary protein levels affect the establishment of intestinal microflora in sika deer during the early wintering period, with higher protein diets promoting greater intestinal microflora development. Alpha diversity indices, including the Shannon index, observed species index, and Chao1 index were higher in the 18% protein groups than in the 15% groups, indicating that a higher protein diet supports a more diverse and complex microbial community in sika deer fawns.

Previous studies reported that the intestinal development in young deer is made dominant by Proteobacteria and Firmicutes, with diversity increasing with age [40]. Bacteroides was the most common in the cecum and colon of young deer, and the proportion of Shigella and Lactobacillus was also higher, which was similar to the results of experiments in the colon and feces of weaned calves [41,42]. Similarly, this study found Firmicutes_A and Bacteroidota as the dominant phyla in the rectal feces of the four groups of early wintering sika deer. Given the known roles of Firmicutes and Bacteroidetes in polysaccharide fermentation, lipid and bile acid metabolism, and energy homeostasis [43,44], these phyla likely exhibit a synergistically symbiotic relationship between Firmicutes and Bacteroidetes, enhancing host’s energy intake, storage, and weight gain in the host.

At the genus level, the dominant microbiota in rectal feces included *Faecousia* and *Cryptobacteroides*. *Faecousia* belongs to the normal microbiota in the intestinal tract of animals and functions as a symbiotic bacterium, promoting the absorption of protein and amino acids. The addition of 60 d Enterococcus faecalis could increase the apparent digestibility of ADF in sheep and regulate the relative abundance of phylum or genus of rumen microflora in sheep [45]. *Cryptobacteroides* contributes to the degradation of complex plant polysaccharides such as cellulose and hemicellulose to short-chain fatty acids [46].

The LDA diagram further highlighted the differences in bacterial composition across the four groups. Notably, the P18E4 group exhibited enrichment of Bacteroidota, while the P18E8 group had significant increases in *Oscillospirales* and *Oscillospiraceae*. *Oscillospira* is widely found in the digestive tracts of herbivores and in the human gut. In the study of human gut microbiota, *Oscillospira* has been associated with carbohydrate fermentation and short-chain fatty acid production [47], although it is also linked to obesity and constipation in certain context [48]. Studies in goats have shown that *Oscilospira* is involved in the host immune maturation process, but the specific function is not yet clear [49]. Therefore, high-protein, high-fat diets may carry potential risks of such conditions in young deer.

In summary, a diet with a protein level of 18% and a fat level of 4% effectively regulates microbiota composition, enhances nutrient absorption, promotes weight gain, and mitigates intestinal diseases in sika deer during the early wintering period.

## 5. Conclusions

This study provides novel evidence that a diet containing 18% protein and 4% fat significantly enhances growth performance, nutrient digestibility, and serum growth hormone content in early wintering sika deer young deer fawns. Furthermore, it positively regulates microbiota composition, contributing to improved nutrient absorption and reduced risks of intestinal disorders. These findings address a critical gap in the nutritional management of sika deer fawns, offering practical recommendations for feed formulation to optimize their health and productivity during the early overwintering period.

## Figures and Tables

**Table 1:** Ingredients and nutrient composition of the experimental diets used in the study (%, air-dried basis).

Items	P18E8	P15E8	P18E4	P15E4
Ingredients (%)				
Corn	29.00	40.00	33.00	46.00
Soybean meal	6.00	1.00	6.00	0.60
Corn germ oil meal	5.80	8.00	5.80	6.60
Distillers dried grains with soluble	9.40	1.20	9.40	1.00
Soybean oil	4.00	4.00	0	0
Molasses	3.00	3.00	3.00	3.00
Alfalfa (CP 17%)	40.00	40.00	40.00	40.00
Calcium bicarbonate·2H_2_O	0.60	0.60	0.60	0.60
Mountain flour	0.30	0.30	0.30	0.30
NaCl	0.50	0.50	0.50	0.50
Baking soda	0.70	0.70	0.70	0.70
Premix ^1^	0.70	0.70	0.70	0.70
Total	100.00	100.00	100.00	100.00
Nutrient levels (% DM)				
RDP	9.25	7.19	9.45	7.18
RUP	9.35	8.32	8.61	8.04
RDP/RUP	0.99	0.86	1.10	0.89
NFE	40.36	44.98	44.67	45.80
GE (MJ/kg DM)	18.48	18.28	18.01	17.63
CP	18.60	15.51	18.06	15.22
EE	8.41	8.06	4.76	4.33
CF	11.58	9.54	10.94	12.47
Ca	0.83	0.73	1.03	0.85
P	0.52	0.47	0.61	0.49
NDF	49.78	47.02	48.81	55.62
ADF	15.81	13.04	14.28	17.25

CP, crude protein; DM, dry matter; RDP, rumen degradable protein; RUP, rumen undegradable protein; NFE, nitrogen free extract; GE, gross energy; EE, ether extract; CF, crude fiber; Ca, calcium; P, phosphorus; NDF, neutral detergent fiber; ADF, acid detergent fiber. ^1^ One kilogram of premix contained the following: VA 8.71 IU, VB1 0.0017 mg, VB_2_ 0.012 mg, VD_3_ 8.9424 IU, VE 0.0149IU, VK_3_ 0.0041 mg, folic acid 0.0004 mg, calcium pantothenate 0.0207 mg, niacin 0.0292 mg, FeSO_4_·H_2_O 0.954 mg, MnSO_4_·H_2_O 0.774 mg, ZnSO_4_·H_2_O 0.648 mg, NaSeO_3_ 0.558 mg, CaHPO_4_ 0.0931 g, CaCO_3_ 0.0823 g.

**Table 2:** Effects of dietary protein and fat levels on growth performance of sika deer fawns in early wintering period (mean ± SE, *n* = 8).

Items		IW, kg	FW, kg	TWG, kg	ADWG, g/d	ADFI, kg/d	F/W, %
Individual treatment means ^1^	P18E8	43.88 ± 1.80	48.61 ± 1.82	4.73 ± 0.38 ^yz^	78.85 ± 6.30 ^yz^	1.69 ± 0.05	22.50 ± 1.95 ^y^
	P15E8	43.03 ± 2.29	48.94 ± 1.91	5.91 ± 0.83 ^xy^	98.57 ± 13.81 ^xy^	1.68 ± 0.06	18.55 ± 1.84 ^y^
	P18E4	42.10 ± 1.69	48.74 ± 1.87	6.64 ± 0.39 ^x^	110.71 ± 6.49 ^x^	1.68 ± 0.04	15.52 ± 0.95 ^y^
	P15E4	40.83 ± 1.59	44.47 ± 1.79	3.64 ± 0.62 ^z^	60.71 ± 10.29 ^z^	1.66 ± 0.04	31.90 ± 4.76 ^x^
Means of main effects ^2^							
Protein level	18%	43.05 ± 1.73	48.67 ± 0.87	5.62 ± 0.41	93.72 ± 3.58	1.69 ± 0.05	19.24 ± 1.56 ^b^
	15%	41.93 ± 1.13	46.71 ± 1.25	4.78 ± 0.20	79.64 ± 6.66	1.67 ± 0.04	25.22 ± 1.49 ^a^
Fat level	8%	43.48 ± 1.37	48.77 ± 0.92	5.28 ± 0.37	88.06 ± 4.89	1.69 ± 0.08	20.65 ± 1.44
	4%	41.46 ± 1.22	46.61 ± 1.15	5.14 ± 0.40	85.71 ± 3.93	1.67 ± 0.06	23.71 ± 2.18
*p*-values	Protein	0.574	0.298	0.125	0.125	0.883	0.031
	Fat	0.296	0.252	0.756	0.756	0.882	0.253
	Interaction	0.911	0.226	0.001	0.001	0.925	<0.001

IW, initial weight; FW, final weight; TWG, total weight gain; ADWG, average daily weight gain; ADFI, average daily feed intake; F/W, weight gain to feed ratio. ^1^ Treatment means represent the average values for 8 tanks per treatment; treatment means followed by a different superscript letter (x, y, z) in the same column are significantly different (*p* < 0.05). ^2^ Main effect means followed by a different superscript letter (dietary protein = lowercase, a, b) in the same column are significantly different (*p* < 0.05).

**Table 3:** Effects of dietary protein and fat levels on nutrients apparent digestibility of sika deer fawns in early wintering period (mean ± SE, *n* = 8).

Items		Nutrients Apparent Digestibility %						
		DM	CP	EE	Ca	P	NDF	ADF
Individual treatment means ^1^	P18E8	75.44 ± 0.88 ^x^	65.08 ± 1.39 ^y^	88.53 ± 0.57 ^w^	39.58 ± 1.12 ^x^	73.37 ± 2.38 ^w^	61.83 ± 2.85 ^x^	18.85 ± 1.34 ^x^
	P15E8	75.65 ± 0.34 ^x^	69.86 ± 1.16 ^x^	89.04 ± 0.56 ^w^	35.53 ± 1.45 ^y^	71.62 ± 1.67 ^w^	60.41 ± 1.96 ^x^	17.53 ± 1.54 ^x^
	P18E4	79.47 ± 0.66 ^w^	80.46 ± 0.58 ^w^	89.18 ± 0.31 ^w^	56.13 ± 0.94 ^w^	75.92 ± 1.55 ^w^	75.10 ± 0.71 ^w^	29.18 ± 1.43 ^w^
	P15E4	72.09 ± 0.16 ^y^	58.14 ± 0.95 ^z^	86.30 ± 0.87 ^x^	30.17 ± 1.72 ^z^	51.86 ± 4.62 ^x^	52.71 ± 1.10 ^y^	14.09 ± 1.01 ^y^
Means of main effects ^2^								
Protein level	18%	77.46 ± 0.18 ^a^	72.77 ± 1.46 ^a^	88.85 ± 0.30 ^a^	47.86 ± 1.40 ^a^	74.65 ± 0.65 ^a^	68.46 ± 0.29 ^a^	24.02 ± 1.11 ^a^
	15%	73.87 ± 0.25 ^b^	64.00 ± 0.71 ^b^	87.67 ± 0.92 ^b^	32.85 ± 0.15 ^b^	61.74 ± 1.94 ^b^	56.56 ± 0.90 ^b^	15.81 ± 0.98 ^b^
Fat level	8%	75.55 ± 0.54	67.47 ± 0.28	88.78 ± 0.57 ^A^	37.56 ± 0.11 ^B^	72.50 ± 0.68 ^A^	61.12 ± 0.82 ^B^	18.19 ± 0.41 ^B^
	4%	75.78 ± 0.52	69.30 ± 1.73	87.74 ± 0.72 ^B^	43.15 ± 1.95 ^A^	63.89 ± 1.60 ^B^	63.90 ± 1.84 ^A^	21.63 ± 1.43 ^A^
*p*-values	Protein	<0.001	<0.001	0.003	<0.001	<0.001	<0.001	<0.001
	Fat	0.598	0.097	0.002	<0.001	0.005	<0.001	<0.001
	Interaction	<0.001	<0.001	0.022	<0.001	<0.001	<0.001	<0.001

DM, dry matter; CP, crude protein; EE, ether extract; Ca, calcium; P, phosphorus; NDF, neutral detergent fiber; ADF, acid detergent fiber. ^1^ Treatment means represent the average values for 8 tanks per treatment; treatment means followed by a different superscript letter (w, x, y, z) in the same column are significantly different (*p* < 0.05). ^2^ Main effect means followed by a different superscript letter (dietary protein = lowercase, a, b; dietary fat = uppercase, A, B) in the same column are significantly different (*p* < 0.05).

**Table 4:** Effects of dietary protein and fat levels on serum biochemical indexes of sika deer fawns in early wintering period (mean ± SE, *n* = 8).

Items		TG, mmol/L	CHO, mmol/L	HDL-C, mmol/L	LDL-C, mmol/L	TP, g/L	ALB, g/L	GLU, mmol/L	Urea, mmol/L
Individual treatment means ^1^	P18E8	0.16 ± 0.02	1.90 ± 0.07 ^z^	1.11 ± 0.06 ^z^	0.27 ± 0.03	59.29 ± 0.41	31.23 ± 0.34	9.31 ± 1.33	10.12 ± 0.38 ^y^
	P15E8	0.18 ± 0.02	2.44 ± 0.10 ^y^	1.56 ± 0.08 ^y^	0.28 ± 0.04	58.94 ± 0.69	30.93 ± 1.16	10.62 ± 0.43	9.65 ± 0.27 ^y^
	P18E4	0.16 ± 0.03	1.74 ± 0.08 ^z^	0.99 ± 0.05 ^z^	0.25 ± 0.03	62.01 ± 1.18	32.39 ± 0.45	10.83 ± 0.34	7.06 ± 0.13 ^z^
	P15E4	0.14 ± 0.01	1.60 ± 0.17 ^z^	0.94 ± 0.11 ^z^	0.22 ± 0.03	57.56 ± 2.04	28.87 ± 2.04	8.68 ± 0.55	10.09 ± 0.47 ^y^
Means of main effects ^2^									
Protein level	18%	0.16 ± 0.03	1.82 ± 0.07	1.05 ± 0.07 ^b^	0.26 ± 0.03	60.65 ± 0.80	31.81 ± 0.52	10.07 ± 0.79	8.59 ± 0.13 ^b^
	15%	0.16 ± 0.03	2.02 ± 0.08	1.25 ± 0.04 ^a^	0.25 ± 0.01	58.25 ± 1.22	29.90 ± 1.18	9.65 ± 0.66	9.87 ± 0.13 ^a^
Fat level	8%	0.17 ± 0.03	2.17 ± 0.06 ^A^	1.34 ± 0.03 ^A^	0.28 ± 0.04	59.12 ± 0.19	31.08 ± 0.24	9.97 ± 0.77	9.89 ± 0.17 ^A^
	4%	0.15 ± 0.02	1.67 ± 0.05 ^B^	0.96 ± 0.04 ^B^	0.23 ± 0.03	59.79 ± 1.12	30.63 ± 0.67	9.76 ± 0.68	8.57 ± 0.17 ^B^
*p*-values	Protein	0.764	0.083	0.026	0.725	0.065	0.194	0.784	<0.001
	Fat	0.284	<0.001	<0.001	0.183	0.112	0.125	0.594	<0.001
	Interaction	0.371	0.004	0.005	0.542	0.596	0.711	0.133	<0.001

TG, triglycerides; CHO, cholesterol; HDL-C, high-density lipoprotein cholesterol; LDL-C, low-density lipoprotein cholesterol; TP, total protein; ALB, albumin; GLU, glucose. ^1^ Treatment means represent the average values for 8 tanks per treatment; treatment means followed by a different superscript letter (y, z) in the same column are significantly different (*p* < 0.05). ^2^ Main effect means followed by a different superscript letter (dietary protein = lowercase, a, b; dietary fat = uppercase, A, B) in the same column are significantly different (*p* < 0.05).

**Table 5:** Effects of dietary protein and fat levels on serum hormonal indexes of sika deer fawns in early wintering period (mean ± SE, *n* = 8).

Items		GH, ng/mL	IGF, ng/mL
Individual treatment means ^1^	P18E8	5.63 ± 0.11 ^y^	119.27 ± 2.68
	P15E8	4.91 ± 0.13 ^z^	117.69 ± 3.86
	P18E4	4.77 ± 0.16 ^z^	114.51 ± 2.84
	P15E4	4.72 ± 0.15 ^z^	114.17 ± 1.68
Means of main effects ^2^			
Protein level	18%	5.20 ± 0.05 ^a^	116.89 ± 1.45
	15%	4.82 ± 0.04 ^b^	115.93 ± 1.94
Fat level	8%	5.27 ± 0.05 ^A^	118.48 ± 1.41
	4%	4.75 ± 0.04 ^B^	114.34 ± 1.65
*p*-values	Protein	0.011	0.745
	Fat	<0.001	0.835
	Interaction	0.024	0.172

GH, growth hormone; IGF, insulin-like growth factor. ^1^ Treatment means represent the average values for 8 tanks per treatment; treatment means followed by a different superscript letter (y, z) in the same column are significantly different (*p* < 0.05). ^2^ Main effect means followed by a different superscript letter (dietary protein = lowercase, a, b; dietary fat = uppercase, A, B) in the same column are significantly different (*p* < 0.05).

**Table 6:** Effects of dietary protein and fat levels on α-indexes of sika deer fawns in early wintering period (mean ± SE, *n* = 8).

Items		Chao1	Observed Species	Shannon
Individual treatment means ^1^	P18E8	2814.07 ± 121.83	2716.21 ± 131.95	10.14 ± 0.16
	P15E8	2541.11 ± 324.36	2447.47 ± 309.52	9.91 ± 0.24
	P18E4	2713.96 ± 222.28	2604.11 ± 206.98	10.10 ± 0.14
	P15E4	2639.61 ± 386.87	2510.05 ± 384.57	9.98 ± 0.37
Means of main effects ^2^				
Protein level	18%	2764.01 ± 180.71	2660.16 ± 177.39	10.12 ± 0.15 ^a^
	15%	2587.46 ± 347.35	2476.92 ± 337.10	9.94 ± 0.30 ^b^
Fat level	8%	2669.56 ± 280.75	2573.94 ± 273.19	10.02 ± 0.23
	4%	2676.78 ± 307.21	2557.08 ± 302.27	10.04 ± 0.27
*p*-values	Protein	0.090	0.070	0.045
	Fat	0.994	0.799	0.863
	Interaction	0.324	0.373	0.503

^1^ Treatment means represent the average values for 8 tanks per treatment. ^2^ Main effect means followed by a different superscript letter (dietary protein = lowercase, a, b) in the same column are significantly different (*p* < 0.05).

## Data Availability

The raw data supporting the conclusions of this article will be made available by the authors on request.
